# Loss of hepatic carboxylesterase 3 prevents the development of MASLD in mice

**DOI:** 10.1016/j.jlr.2025.100887

**Published:** 2025-08-25

**Authors:** Zaid Batayneh, Xiaoli Pan, Raja Gopoju, Shuwei Hu, Shaoyu Chen, Jiayou Wang, Hui Wang, Lakshitha Gunawardana, Takhar Kasumov, Yanqiao Zhang

**Affiliations:** 1 Department of Biomedical Sciences, Northeast Ohio Medical University, Rootstown, OH, USA; 2 Department of Internal Medicine and the Translational Cardiovascular Research Center, University of Arizona College of Medicine - Phoenix, Phoenix, AZ, USA; 3 Department of Pharmaceutical Sciences, Northeast Ohio Medical University, Rootstown, OH, USA

**Keywords:** MASLD, VLDL, liver, carboxylesterase 3, de novo lipogenesis

## Abstract

Carboxylesterases (CESs) are essential for metabolizing compounds with ester, thioester, and amide bonds. While the roles of CES1 and CES2 in lipid metabolism have been well established, little is known about the role of CES3 in lipid metabolism or metabolic dysfunction–associated steatotic liver disease (MASLD). Here, we report the localization and nutritional regulation of CES3 and its role in MASLD development in mice. CES3 is expressed exclusively in the liver and localizes to the ER. Hepatic CES3 is reduced in patients with metabolic dysfunction–associated steatohepatitis and mice fed a Western diet. Unexpectedly, loss of CES3 alleviates Western diet–induced MASLD, whereas liver-specific overexpression of human CES3 worsens Western diet-induced MASLD. Mechanistically, loss of CES3 reduces de novo lipogenesis and promotes the secretion of VLDL-triglycerides. Thus, the current study has identified a novel role of CES3 in hepatic lipid metabolism and MASLD.

Metabolic dysfunction–associated steatotic liver disease (MASLD) is defined as the presence of excessive triglyceride (TG) storage in the presence of at least one cardiometabolic risk factor and absence of alcohol abuse (1). MASLD comprises metabolic dysfunction–associated steatotic liver and metabolic dysfunction–associated steatohepatitis (MASH), which may further progress to cirrhosis and hepatocellular carcinoma (1, 2). The global prevalence of MASLD is estimated to be 32% (3). Therefore, MASLD is the primary cause of chronic liver disease. MASLD imposes a considerable economic burden, exceeding $100 billion in the United States alone (4). The multifaceted nature of MASLD necessitates identifying the underlying mechanisms and new therapeutic targets.

Carboxylesterases (CESs) belong to at least five gene families and participate in xenobiotic, drug, and lipid metabolism (5). CES1 and CES2 have been extensively investigated for their roles in prodrug metabolism and are also shown to possess TG hydrolase activity in mice, including mouse Ces1d (mouse ortholog of human CES1), Ces1g, and Ces2c (previously mouse Ces2) (6, 7). Interestingly, hepatic Ces2c deficiency promotes liver steatosis (7) whereas loss of hepatic Ces1d (formerly called Ces3/triglyceride hydrolase [TGH]) increases hepatic TG accumulation in chow-fed mice but has no effect on hepatic steatosis in Western diet–fed mice due to its role in fatty acid oxidation (FAO) and VLDL secretion (8).

In mice, there are 7–8 *Ces1* or *Ces2* genes (5), each of which likely shares compensatory effects. In contrast, mice have only two *Ces3* genes, *Ces3a* (*Es31*) and *Ces3b (Es31L)* (5). Humans have only one *CES3* gene. Human CES3 has 45% and 67% amino acid identities with mouse Ces3a and Ces3b, respectively. Compared to CES1 or CES2, little is known about the substrate specificity of CES3, likely due to a low catalytic efficiency (9). So far, the function of CES3 in lipid metabolism is unknown. In this study, we overexpressed human CES3 in the liver of WT mice and used *Ces3*^−/−^ mice to understand the role of CES3 in MASLD. Our results show that the loss of CES3 prevents the development of MASLD.

## Materials and methods

### Mice, diets, and human tissues

C57BL/6J mice (stock #000664) were purchased from The Jackson Laboratory (Bar Harbor, Maine). Heterozygous *Ces3*^+/−^ mice were generated on a C57BL/6 background by Biocytogen through the deletion of both *Ces3a* and *Ces3b* genes on chromosome 8 (∼45 kb) via CRISPR/Cas9 with only exon 1 of *Ces3a* retained. Homozygous *Ces3*^*+/+*^ (control) and *Ces3*^−/−^ mice were generated by crossbreeding *Ces3*^+/−^ mice. The primer sequences for genotyping are WT-F: CCACCATGACCAGGAAGTTGACCTC; KO-F: TACAGGTGTGGTATATCCCGACTGGG; and common-R: GGCTTAGATGCCTACCTGGGGATGA. All mice were housed in a temperature- and humidity-controlled room with a 12-h light/12-h dark cycle and had free access to water and food. The regular chow diet containing 6.5% fat was purchased from LabDiets (stock #5008). The Western diet (42% kcal from fat/0.2% cholesterol) was purchased from Envigo (Cat#TD.88137). The high fat/cholesterol/fructose diet containing 21% fats, 0.21% cholesterol, and 32% fructose was purchased from Research Diets (Cat #D16051004). For the feeding studies, 8- to 10-week-old male mice were randomly allocated and fed either a Western diet or regular chow for 16 weeks. Unless otherwise stated, male mice were used and fasted for 5–6 h before euthanasia. Deidentified human liver samples were obtained from the Liver Tissue Cell Distribution System at the University of Minnesota, and the clinical characteristics of these human subjects have been published previously (10). All the animal experiments were approved by the Institutional Animal Care and Use Committee at Northeast Ohio Medical University. The use of human tissues was approved by the Institutional Review Board at Northeast Ohio Medical University.

### Adeno-associated viruses

The human CES3 coding sequence was cloned into an Adeno-associated virus (AAV) vector under the control of a mouse albumin promoter to generate AAV-ALB-hCES3. AAV8-ALB-Null (control) and AAV8-ALB-hCES3 were produced and titrated by Vector BioLabs. Mice were intravenously injected with 3 × 10^11^ genome copies of AAVs before being placed on a chow or Western diet.

### Quantitative real-time PCR

Total RNA was isolated using TRIzol Reagent (Thermo Fisher Scientific). The mRNA levels were determined by quantitative reverse-transcription polymerase chain reaction on a 7500 real-time PCR machine from Applied Biosystems (Foster City, CA) by using SYBR Green Supermix (Roche, Indianapolis, IN). Relative mRNA levels were quantified using the 2ˆ^-ΔΔCt^ method and normalized to *36b4*.

### Western blot assays

Western blot assays were performed using whole liver lysate extracts of the liver samples or isolated fractions as described [24]. The gel band intensity was quantified using the ImageJ software and normalized to the relevant loading controls. The antibody against CES3 (cat #GTX66446; 1:1000 dilution) was purchased from GeneTex. The antibodies against CES1 (cat #ab45957; 1:1000 dilution), CES2 (cat #ab56528; 1:1000 dilution), or tubulin (cat #ab4074; 1:2000 dilution) were purchased from Abcam. The antibody against ATGL (cat #2439; 1:1000 dilution) was purchased from Cell Signaling. The antibodies against SREBP-1 (cat #NB600-582; 1:1000 dilution) or calnexin (cat #NB100-1965; 1:1000 dilution) were purchased from Novus. The MTP antibody (cat #sc135994; 1:1000 dilution) was purchased from Santa Cruz Biotechnology. The APOB antibody (cat #20578-1-AP; 1:1000 dilution) was purchased from Proteintech.

### Plasma and liver biochemistry

Plasma alanine aminotransferase (ALT), aspartate aminotransferase (AST), TG, and total cholesterol levels were determined using Infinity reagents from Thermo Fisher Scientific. To measure hepatic lipid levels, approximately 100 mg of liver tissue was homogenized in methanol, and lipids were extracted using chloroform/methanol (2:1 v/v) as described (11). Hepatic TG or total cholesterol levels were quantified using Infinity reagents (Thermo Fisher Scientific). Hepatic total FFAs were determined using a kit from Wako Chemicals USA (Richmond, VA).

### Hepatic fatty acid composition

Hepatic fatty acid composition was analyzed by GC-MS as previously described (7). Briefly, liver tissue samples were spiked with 50 nmol of C17:0 fatty acid, and lipids were extracted (11). After evaporating the solvents under nitrogen gas, the residue was dissolved in 2 ml of a methanol-benzene mixture (v/v, 4:1). Acetyl chloride (200 μl) was then slowly added under agitation for 1 min. Tightly closed samples were heated at 100°C for 1 h to facilitate the methanolysis of fatty acids. After cooling to room temperature, the reaction was quenched with 2.5 ml of 12% potassium carbonate solution. The upper benzene fraction was removed, and methyl esters of fatty acids were extracted using 1 ml of benzene. The combined benzene extract was evaporated, and the residue was reconstituted in 100 μl of ethyl acetate before GC-MS analysis. Analyses of the methyl esters of fatty acids were performed on an Agilent 5977A mass spectrometer equipped with a 7890 gas chromatograph, an autosampler, and an HP-5MS fused silica capillary column (30 m, 250 μm i.d., 0.25 μm film thickness), analyzed by electron impact ionization GC-MS. The injector temperature was 290°C, and the transfer line temperature was 300°C. The GC temperature program was initiated at 80°C, held for 1 min, then increased by 10°C/min to 270°C, followed by a 45°C/min increase to 310°C, and finally held for 10 min. The ion source was set at 250°C and the quadrupole at 150°C. The methyl esters of the analyzed fatty acids were eluted between 12 min and 20 min. Under electron impact ionization, ions (*m/z*) were monitored: 242.2 (C14), 227.2 (C16), 236.2 (C16:1), 241.2 (C17), 255.2 (C18), 264.3 (C18:1), and 294.3 (C18:2). Fatty acid species were quantified using calibration curves and the ratios of the integrated peak areas of fatty acid species and C17:00 fatty acid internal standard.

### Oil red O and H&E staining

Liver tissues were fixed in 10% formalin and then embedded in OCT or paraffin. The liver sections were stained with oil red O (ORO) or H&E. Hepatic neutral lipid accumulation and morphology were determined using images acquired by an Olympus microscope.

### Isolation of cytoplasm, ER, and microsomes

The ER was isolated using an ER isolation kit (cat # ER0100, Sigma), and modifications were made as described (12). In brief, the liver was sliced, washed with 10 ml of PBS, and homogenized with a polytetrafluoroethylene pestle in a glass tube homogenizer using the extraction buffer, which consisted of 100 ml of 50 mM Hepes (pH 7.8), 1.25 M sucrose, 5 mM EGTA, and 125 mM KCl. Next, the mixture was centrifuged at 1,000 *g* for 10 min to remove cell debris and nuclei. The thin floating lipid layer was removed, and the supernatant, referred to as the postnuclear fraction, was transferred to a new tube. Afterward, the postnuclear fraction was centrifuged at 12,000 *g* to remove mitochondria and lipids, resulting in a crude ER supernatant. Next, the supernatant was centrifuged for 60 min at 100,000 *g* in an ultracentrifuge at 4°C. The pellet obtained was the ER fraction. The middle layer was the cytoplasm, and the upper layer is the lipid droplet fraction. The microsomes were isolated using an isolation kit (cat #ab206995) purchased from Abcam.

### Body composition and energy expenditure

The EchoMRI-700 (EchoMRI LLC, Houston, TX) was used to measure the whole-body fat and lean masses of the mice. The comprehensive laboratory animal monitoring system was used to measure oxygen consumption and heat production as described (13, 14). The data were analyzed using the web-based CalR program (15).

### VLDL-TG secretion

VLDL-TG secretion was determined as described (16). In brief, mice were fasted for 5 h, followed by an intravenous injection of tyloxapol (500 mg/kg) to inhibit lipoprotein lipase activity. Blood was collected from the retro-orbital cavities at various time points (0, 30, 60, 90, 120, and 180 min), and TG levels were quantified in plasma using an Infinity reagent. VLDL-TG secretion rate was calculated using slopes as described (17).

### TGH activity assay

Hepatic whole-cell lysate (WCL) proteins and microsomal proteins were isolated, and the TGH activity was measured using 1.6 μCi ^3^H-triolein as a substrate as described (7). In brief, the liver was harvested after a 6-h fast, homogenized in a lysis buffer, and then centrifuged at 800 *g* to remove cell debris. The supernatant was then centrifuged at 100,000 *g* for 1 h. The WCL and microsome portions were used separately for the TGH assay. One liver extracts or microsomes (100 μg of protein) were incubated at 37°C with 100 μl substrates containing 0.15 mM cold triolein, 0.32 μM [^3^H]triolein, 10 μM egg yolk lecithin, 100 μM sodium taurocholate, 1 mM dithiothreitol, and 50 mM potassium phosphate (pH 7.4). After 1 h, the reaction was stopped by adding 3.75 ml of a methanol/chloroform/heptane (10:9:7) mixture and 1 ml of 0.1 M potassium carbonate. After centrifuging at 800 *g*, 1 ml of the top phase was used to count radioactivity using a liquid scintillation counter.

### Hepatic de novo lipogenesis

On day 0 (10 days before sacrifice), 60 μl of baseline blood samples were collected. On day 3, the mice received an intraperitoneal bolus of ^2^H_2_O, consisting of about 30 μl of 99.99% ^2^H_2_O-labeled saline per gram of body weight, followed by an 8% ^2^H_2_O solution in their drinking water. Blood samples of approximately 60 μl were collected from the retro-orbital sinus on days 4, 5, and 7. On day 10 (the day of sacrifice), mice were sacrificed and about 1 ml was collected from the retro-orbital sinus. Palmitate synthesis was analyzed by GC-MS as described (18).

### Statistical analysis

All data were expressed as mean ± SEM. Statistical significance was analyzed using an unpaired Student *t* test or ANOVA for multiple comparisons, as determined by Prism (GraphPad, CA). Differences were considered statistically significant at *P* < 0.05.

## Results

### CES3 is expressed in the liver and localized to the ER

**Fig. 1:**
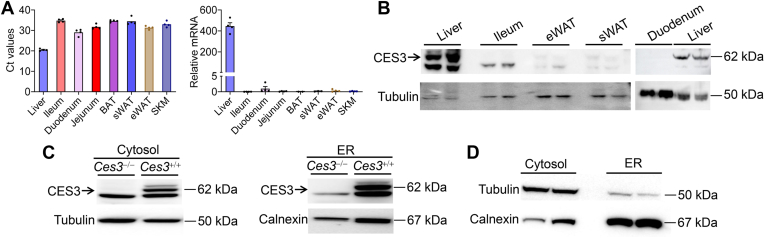
CES3 is expressed in the liver and localized to the ER. A-B: *Ces3 Ct* values (A, left panel), relative *Ces3a/b* mRNA levels after normalization to *36b4* (A, right panel), and CES3 protein levels (B) in mouse tissues. C: CES3 expression in cytosol and ER. D: The purity of cytosol and ER fractions was assessed using specific markers (D) Data are expressed as mean ± SEM. BAT, brown adipose tissue; CES, carboxylesterase; eWAT, epididymal white adipose tissue; SKM, skeletal muscle; sWAT, subcutaneous white adipose tissue.

So far, little is known about the tissue distribution or function of CES3. To investigate the tissue distribution of CES3 in mice, we measured *Ces3* Ct values using quantitative real-time PCR in the liver, small intestine, white adipose tissue, brown adipose tissue, and skeletal muscle tissues of 10-week-old C57BL/6 mice, and also assessed CES3 protein expression using Western blotting. The data of Figure 1A show that *Ces3a/b* mRNA was present >450-fold more than in any other tissues. Western blotting shows that CES3 protein was undetectable in other tissues. Thus, mouse *Ces3* is expressed exclusively or predominantly in the liver.

To determine the subcellular localization of CES3, we isolated cytosol and ER from *Ces3*^+/+^ mice or *Ces3*^−/−^ mice (control). Figure 1C shows that CES3 was present in cytosol and ER. The purity of these organelles is presented in Figure 1D, which shows incomplete separation of these fractions. CES3 has a signal sequence directing the protein to the lumen of ER. The cytosolic presence of CES3 may be due to the contamination of the ER during isolation. Thus, the data of Figure 1 indicate that CES3 expression is restricted to the liver and localizes to ER.

### Hepatic CES3 is markedly reduced in MASH patients and mice fed a Western diet

**Fig. 2:**
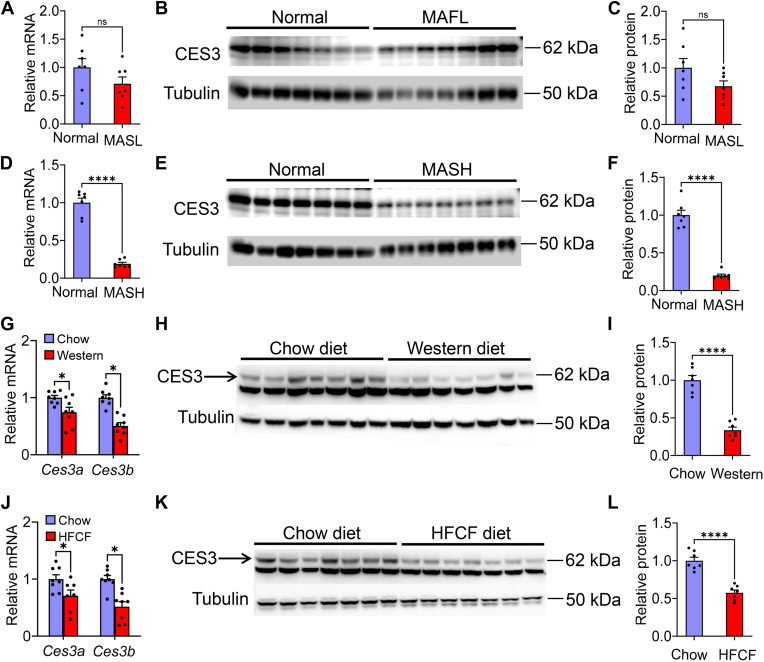
Hepatic CES3 is reduced in MASH patients and Western diet–fed mice. A-F: Hepatic *CES3* mRNA (A, D) or protein (B-C, E-F) levels in MASL (A-C) or MASH (D-F) patients (n = 7). G-I: C57BL/6 mice were fed a regular chow or Western diet for 16 weeks (n = 7). Hepatic *Ces3a/b* mRNA (G) and protein (H-I) levels were determined. J-L: C57BL/6 mice were fed a regular chow or HFCF diet for 16 weeks (n = 7). Hepatic *Ces3a/b* mRNA (J) and protein (K-L) levels were determined. ns, not significant. ∗*P* < 0.05, ∗∗∗*P* < 0.001, and ∗∗∗∗*P* < 0.0001. CES, carboxylesterase; HFCF, high fat/cholesterol/fructose; MASH, metabolic dysfunction–associated steatohepatitis; MASL, metabolic dysfunction–associated steatotic liver.

In MAFL patients, hepatic *CES3* mRNA and protein levels tended to decrease (Fig. 2A–C). In MASH patients, hepatic *CES3* mRNA and protein levels were reduced by >80% (Fig. 2D–F). In mice fed a Western diet for 16 weeks, hepatic *Ces3a* and *Ces3b* mRNA levels were significantly reduced (Fig. 2G) and hepatic CES3 protein levels were reduced by 66% (Fig. 2H, I). Similarly, in mice fed a high fat/cholesterol/fructose diet for 16 weeks, hepatic *Ces3a* and *Ces3b* mRNA levels were significantly reduced (Fig. 2J), and hepatic CES3 protein levels were reduced by >43% (Fig. 2K–L). These data demonstrate that hepatic CES3 is reduced in MASH patients and mouse models of MASLD.

### Loss of CES3 improves Western diet–induced MASLD

**Fig. 3:**
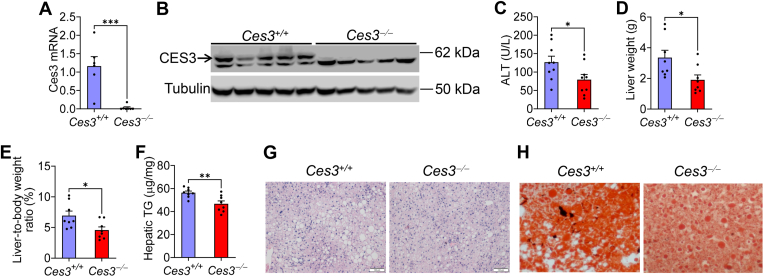
Loss of CES3 improves Western diet-induced MASLD. 8–10 weeks *Ces3*^+/+^ and *Ces3*^−/−^ mice were fed a Western diet for 16 weeks (n = 10 per group). A: Hepatic *Ces3* mRNA levels. B: Hepatic CES3 protein was absent in *Ces3*^−/−^ mice (top band). C: Plasma ALT levels. D: Liver weight. E: Liver-to-body weight ratio (%). F: Hepatic TG levels. G: H&E staining of liver sections. H: Oil red O staining of liver sections. Scale bars in (G): 100 μm. Data are expressed as mean ± SEM. Statistical analysis was performed using a two-tailed, unpaired *t* test (A, C-F). ∗*P* < 0.05 and ∗∗*P* < 0.01. ALT, alanine aminotransferase; CES, carboxylesterase; MASLD, metabolic dysfunction–associated steatotic liver disease; TG, triglyceride.

To understand the role of CES3 in lipid metabolism, we generated *Ces3*^−/−^ mice by deleting both *Ces3a* and *Ces3b* genes using CRISPR/Cas9 (see supplemental Fig. S1A–C). On a regular chow diet, there was no change in body weight, liver weight, liver-to-body weight ratio, hepatic TGs, plasma TG, or plasma cholesterol (supplemental Fig. S1D–I). When *Ces3*^*+/+*^ mice and *Ces3*^*−/−*^ mice were fed a Western diet for 16 weeks (Fig. 3A, B), there was a 38% reduction in plasma ALT levels (Fig. 3C), 44% reduction in liver weight (Fig. 3D), 34% reduction in liver-to-body weight ratio (Fig. 3E), and 18% reduction in hepatic TG levels (Fig. 3F) in *Ces3*^−/−^ mice. Histological staining with H&E (Fig. 3G) or ORO (Fig. 3H) showed reduced neutral lipid accumulation in the liver of *Ces3*^−/−^ mice.

Over the course of 16-week Western diet feeding, there was no significant changes in body weight, body fat mass, or body fat content (supplemental Fig. S2A–C), which was consistent with unchanged energy expenditure (supplemental Fig. S2D). In addition, loss of *Ces3* did not alter plasma AST, TG or cholesterol levels (supplemental Fig. S2E–G), or hepatic cholesterol or FFA levels (supplemental Fig. S2H, I). Taken together, our data demonstrate that loss of CES3 prevents Western diet–induced MASLD.

### Loss of CES3 inhibits de novo lipogenesis

**Fig. 4:**
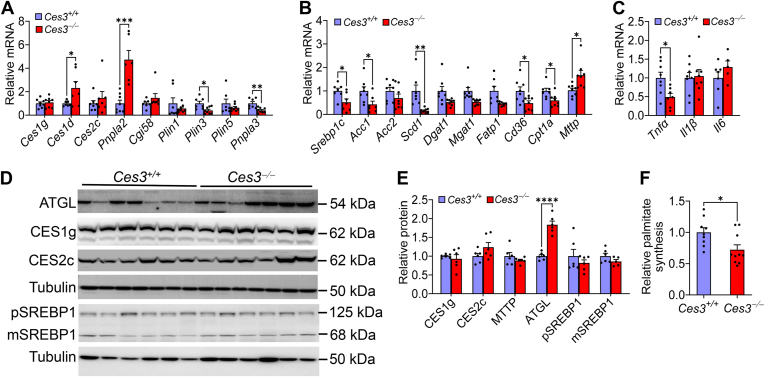
Loss of CES3 inhibits lipogenesis and inflammatory genes in Western diet–fed mice. 8–10 weeks *Ces3*^+/+^ and *Ces3*^−/−^ mice were fed a Western diet for 16 weeks (n = 10 per group). A-C: Hepatic mRNA levels. D-E: Hepatic protein levels were analyzed by Western blotting (D) and then quantified (E). F: Hepatic de novo synthesis of palmitate was analyzed by GC-MS. Data are expressed as mean ± SEM. Statistical analysis was performed using a two-tailed, unpaired *t* test (A-C, E-F). ∗*P* < 0.05, ∗∗*P* < 0.01, ∗∗∗*P* < 0.001, and ∗∗∗*P* < 0.0001. CES, carboxylesterase.

In the liver, loss of *Ces3* did not affect mRNA levels of *Ces1g*, *Ces2c*, comparative gene identification-58, perilipin 1 (*Plin1*), or *Plin5*, but significantly induced *Ces1d* and adipose TG lipase (*Pnpln2*) and repressed *Plin3* and patatin-like phospholipase domain–containing protein 3 (Fig. 4A). Loss of *Ces3* also significantly inhibited hepatic mRNA levels of lipogenic genes, including *Srebp1c*, acetyl-CoA CES 1 (*Acc1*), and stearoyl-CoA desaturase 1 (*Scd1*), and genes involved in fatty acid uptake (gene cluster of differentiation 36 [*Cd36*]) or FAO (carnitine palmitoyltransferase 1 (*Cpt1a*)) while inducing microsomal triglyceride transfer protein (*MTTP*) (Fig. 4B). In addition, loss of *Ces3* significantly reduced hepatic *Tnfα* mRNA levels (Fig. 4C). At protein levels, loss of *Ces3* significantly induced ATGL but had no impact on CES1, CES2, MTTP, or SREBP1 expression (Fig. 4D, E). In line with the reduced lipogenic genes, hepatic de novo synthesis of palmitate was significantly reduced (Fig. 4F), indicating that loss of *Ces3* inhibits de novo lipogenesis (DNL).

### Overexpression of hepatic CES3 aggravates Western diet–induced hepatosteatosis

**Fig. 5:**
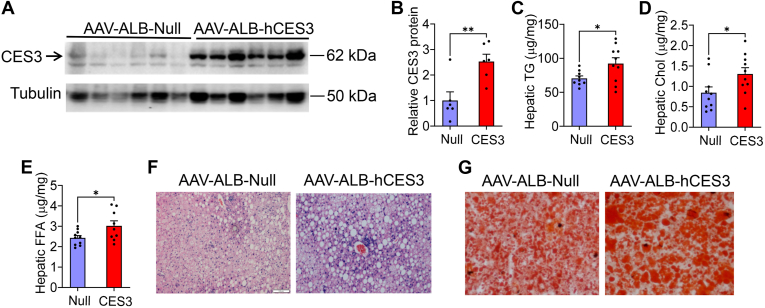
Overexpression of hepatic CES3 aggravates Western diet–induced MASLD. Eight- to ten-week old C57BL/6 mice were i.v. injected with either AAV-ALB-Null or AAV-ALB-hCES3 and then fed a Western diet for 16 weeks (n = 10 per group). A-B: Western blot analysis of hepatic CES3 protein levels. C: Hepatic TG levels. D: Hepatic cholesterol (chol) levels. E: Hepatic FFA levels. F-G: H&E (F) or oil red O (G) staining of liver sections. Scale bars in (F): 100 μm. Statistical analysis was conducted using a two-tailed, unpaired *t* test (B-E). ∗*P* < 0.05 and ∗∗*P* < 0.01. AAV, adeno-associated virus; CES, carboxylesterase; MASLD, metabolic dysfunction–associated steatotic liver disease; TG, triglyceride.

To investigate whether gain of hepatic CES3 expression has an opposite effect on hepatic lipid metabolism as observed in *Ces3*^−/−^ mice, we generated an AAV overexpressing human CES3 under the control of an albumin promoter (AAV8-ALB-hCES3). On a regular chow diet, overexpression of hepatic CES3 had no effect on hepatic TG, cholesterol, or FFA levels (supplemental Fig. S3A–D). On a Western diet, overexpression of hepatic CES3 significantly increased hepatic TG, cholesterol, and FFA levels (Fig. 5A–E), which was confirmed by histological staining with H&E or ORO (Fig. 5F, G). Overexpression of hepatic CES3 did not change body weight, liver weight, or plasma TG, ALT, or AST levels, but increased plasma cholesterol levels (supplemental Fig. S4A–F).

At mRNA levels, overexpression of hepatic CES3 induced *Srebp1c* but repressed *Pnpln2* expression without affecting other genes involved in lipid metabolism or inflammation, including *Ces1g*, *Ces2c*, *Plin2*, *Plin3*, *Acc1*, *Acc2*, *Scd1*, *Dgat1*, *Dgat2*, *Mgat1*, *Fabp1*, *Cd36*, *Cpt1a*, *Mttp*, *Apob*, *Tnfα*, *Il1β*, *or Il6* (supplemental Fig. S4G, H). Interestingly, hepatic SREBP1 protein levels did not change (Fig. 4I, J). Thus, overexpression of hepatic CES3 aggravates Western diet–induced hepatosteatosis.

### CES3 does not regulate hepatic TGH activity but modulates hepatic fatty acid composition

**Fig. 6:**
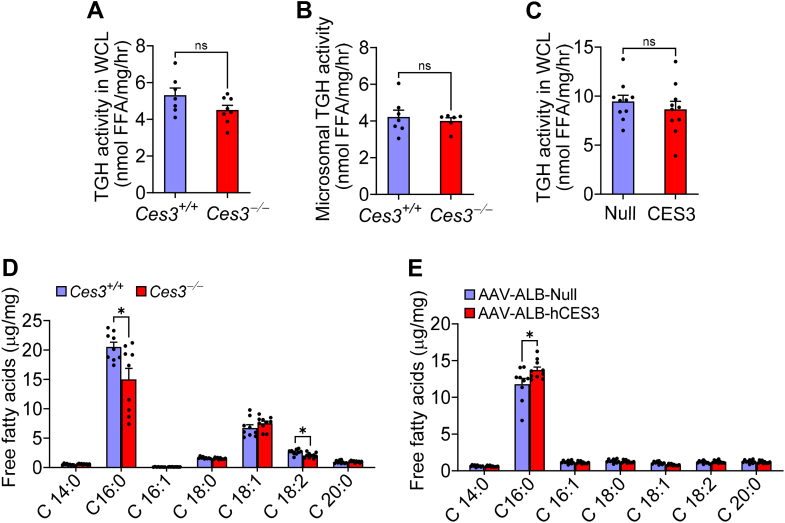
Hepatic CES3 does not regulate triglyceride hydrolase activity but modulates hepatic fatty acid composition. Mice have been described in the legend of Figure 3 or Figure 5. Triglyceride hydrolase activity in whole-cell lysates (WCLs) (A, C) or microsomes (B) were determined. D-E: Hepatic fatty acid composition was analyzed by GC-MS. ns, not significant. Data are presented as mean ± SEM. Statistical analysis was performed using a two-tailed, unpaired *t* test. ∗*P* < 0.05. CES, carboxylesterase.

The induction of ATGL and Ces1d in *Ces3*^−/−^ mice (Fig. 4) implies that hepatic TGH activity may be increased. Surprisingly, loss of CES3 did not alter hepatic TGH activity when WCL or microsomes were used (Fig. 6A, B). Similarly, although mice overexpressing hepatic CES3 had reduced *Atgl* expression (supplemental Fig. S4G), hepatic TGH activity did not change (Fig. 6C).

Consistent with the role of loss of *Ces3* in repressing DNL (Fig. 4), hepatic C16:0 and C18:2 fatty acids were significantly reduced in *Ces3*^−/−^ mice (Fig. 6D). In contrast, overexpression of hepatic CES3 induced C16:0 fatty acids (Fig. 6E). Together, the data of Figure 6 indicate that CES3 does not regulate hepatic TGH activity but modulates hepatic fatty acid composition.

### Loss of CES3 increases VLDL-TG secretion

**Fig. 7:**
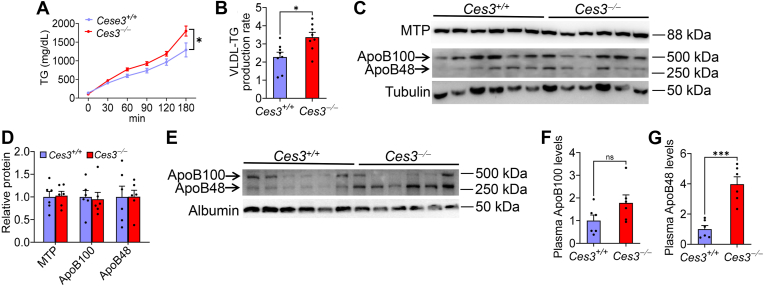
Loss of CES3 increases VLDL-TG secretion. *Ces3*^+/+^ and *Ces3*^−/−^ mice were fed a Western diet for 5 weeks (n = 10 per group). A-B: VLDL-TG secretion over 180 min after i.v. injection of tyloxapol (A) and VLDL-TG production rate (B). C-D: Hepatic protein levels. E-G: Plasma protein levels were analyzed by Western blot assays (E) and plasma ApoB100 (F) or ApoB48 (G) levels were quantified. Data are expressed as mean ± SEM. ns, not significant. Statistical analysis was conducted using two-way ANOVA (A) or a two-tailed, unpaired *t* test (B, D, F-G). ∗*P* < 0.05, and ∗∗∗*P* < 0.001. CES, carboxylesterase; TG, triglyceride.

VLDL secretion plays an important role in regulating hepatic TG accumulation. Interestingly, loss of *Ces3* increased hepatic VLDL-TG secretion (Fig. 7A) with a 47% increase in VLDL-TG secretion rate (Fig. 7B). There was no change in hepatic ApoB or MTP levels (Fig. 7C, D). Consistent with the increased VLDL secretion, plasma ApoB100 levels tended to increase (Fig. 7E, F), while plasma ApoB48 levels were increased by > 4-fold (Fig. 7E, G). Thus, *Ces3*^−/−^ mice have increased VLDL-TG secretion, which may contribute to the reduction in hepatic TG levels in these mice.

## Discussion

So far, the role of CES3 in lipid metabolism has not been elucidated. Our data suggest that the expression of mouse *Ces3a/b* is restricted to the liver. In MASH patients and mouse models of MASLD, hepatic CES3 is markedly reduced. Unexpectedly, we find that genetic loss of mouse *Ces3a/b* prevents Western diet–induced MASLD. Such an unexpected finding is supported by our gain-of-function study showing that overexpression of human CES3 in mice aggravates Western diet–induced MASLD. Thus, CES3 promotes the development of MASLD. Such a finding appears contradictory to the reduced CES3 expression in MASLD. It may be plausible to speculate that during Western diet feeding or the development of MASLD, the liver reduces CES3 expression in order to prevent lipid accumulation, but such a reduction is insufficient to overcome the development of MASLD caused by overall metabolic changes in this process.

The rate of TG synthesis, lipolysis, and secretion determines hepatic TG levels. Our mechanistic studies show that genetic loss of CES3 prevents MASLD likely via inhibiting DNL and inducing VLDL-TG secretion. Hepatic lipogenic genes (*Acc1/2*, *Scd1*) and DNL are reduced in *Ces3*^−/−^ mice. Interestingly, hepatic precursor or nuclear SREBP1 protein levels do not change although *Srebp1c* mRNA level is reduced in *Ces3*^−/−^ mice, implying the SREBP1-independent regulation of DNL genes. The rate of hepatic VLDL-TG is significantly increased in *Ces3*^−/−^ mice, suggesting that the increased VLDL-TG secretion likely contributes to the reduced TG accumulation in *Ces3*^−/−^ mice. *Ces3* deficiency does not regulate hepatic MTP or ApoB protein levels, implying that *Ces3* deficiency stimulates VLDL-TG secretion via yet-to-be-determined mechanisms.

Hepatic FAO and uptake are known to play an important role in regulating hepatic TG levels. CPT1A is the key rate-limiting enzyme of FAO, whereas CD36 plays an important role in hepatic fatty acid uptake. Although hepatic *Cd36* and *Cpt1a* are reduced in *Ces3*^−/−^ mice, they do not change in mice overexpressing hepatic CES3. These findings suggest that neither hepatic fatty acid uptake nor FAO plays an important role in mediating CES3’s effect on hepatic TG levels.

Previous studies have shown that loss of Ces1d decreases VLDL-TG secretion, but represses DNL and increases FAO and insulin sensitivity, leading to protection of hepatosteatosis in two MASH mouse models (19). Ces1d is proposed to mobilize luminal TG for VLDL assembly (6). However, how the loss of Ces1d inhibits DNL and induces FAO remains unknown. In contrast, loss of Ces1g or Ces2c aggravates hepatosteatosis, whereas their overexpression has opposite effects (6, 7, 20, 21, 22). Ces1g or Ces2c inhibits DNL likely resulting from their TGH activity, which releases PUFAs to inhibit lipogenic genes (6, 7). Unlike Ces1d, Ces1g, and Ces2c, which possess TGH activity (6), CES3 does not appear to have TGH activity. However, the findings that Ces1d and ATGL are induced in *Ces3*^−/−^ mice, whereas ATGL is reduced in mice overexpressing hepatic CES3, suggest that CES3 may possess hydrolase activity for other substrates. It will be interesting to examine whether CES3 can hydrolyze or modify other lipid species or substrates, for example, diacylglyceride, monoacylglyceride, and phospholipids. Phospholipids are an important component of VLDL and cellular membranes, and regulate lipid droplet formation, and DNL (23). Thus, CES3 may regulate DNL and VLDL-TG secretion by modifying other lipid species.

In summary, we have identified a novel role of CES3 in hepatic lipid metabolism and MASLD. Although the exact mechanism underlying the regulation of hepatic TG metabolism remains to the further explored, our data suggest that targeting CES3 may represent a new approach to treat MASLD.

## Data availability

The data are available upon request.

## Supplemental data

This article contains supplemental data.

## Conflict of interest

The authors declare that they have no conflicts of interest with the contents of this article.
