# Supplementary material for: Loss of hepatic carboxylesterase 3 prevents the development of MASLD in mice
Source: J Lipid Res. 2025 Aug 25;66(10):100887. doi: 10.1016/j.jlr.2025.100887 (PMC12481058; doi:10.1016/j.jlr.2025.100887)
Supplement: Supplementary Figures [file mmc1.pdf]

Supplementary Information

Supplementary Figure 1. Generation and characterization of *Ces3*$^{-/-}$ mice fed a regular chow diet. (A) Diagram illustrating the strategy for generating *Ces3*$^{-/-}$ mice using CRISPR/Cas9. (**B**) PCR genotyping of *Ces3*$^{+/+}$, *Ces3*$^{+/-}$, and *Ces3*$^{-/-}$ mice. (**C-I**) *Ces3*$^{+/+}$ and *Ces3*$^{-/-}$ mice were maintained a regular chow diet for 10 weeks (n=10 per group). Hepatic protein levels were analyzed by Western blot assays (**C**). Body weight (**D**), liver weight (**E**), liver-to-body weight ratio (**F**), hepatic TG (**G**), plasma TG (**H**) and plasma cholesterol (**I**) levels were determined. ns, not significant.

Supplementary Figure 2. Changes in Western diet-fed *Ces3*$^{-/-}$ mice. *Ces3*$^{+/+}$ and *Ces3*$^{-/-}$ mice were fed a Western diet for 16 weeks (n=10 per group). (**A**) Body weight. (**B**) Body fat mass. (**C**) Body fat content. (**D**) Energy expenditure. (**E**) Plasma AST levels. (**F**) Plasma TG levels. (**G**) Plasma cholesterol levels. (**H**) Hepatic cholesterol levels. (**I**) Hepatic FFA levels. ns, not significant. Repeated two-way ANOVA (**A-C**), ANCOVA (**D**), or 2-tailed, unpaired t-test (**E-I**) was performed.

Supplementary Figure 3. Changes in regular chow diet-fed C57BL/6 mice overexpressing hepatic CES3. C57BL/6 mice were i.v. injected with either AAV-ALB-Null or AAV-ALB-hCES3 and then fed a regular chow diet for 10 weeks (n=10 per group). (**A**) Hepatic protein levels. (**B**) Hepatic TG levels. (**C**) Hepatic total cholesterol levels. (**D**) Hepatic FFA levels. Statistical analysis was performed using a 2-tailed, unpaired t-test (**B-D**). *P<0.05

Supplementary Figure 4. Figure 4. Changes in Western diet-fed C57BL/6 mice overexpressing hepatic CES3. C57BL/6 mice were i.v. injected with either AAV-ALB-Null or AAV-ALB-hCES3 and then fed a Western diet for 10 weeks (n=10 per group). (**A**) Body weight. (**B**) Liver weight. (**C**) Plasma TG levels. (**D**) Plasma cholesterol levels. (**E**) Plasma ALT levels. (**F**) Plasma AST levels. (**G-H**) Hepatic mRNA levels. (**I**) Analysis of hepatic protein levels by Western blotting. (**J**) Hepatic precursor and mature SREBP1 protein levels. Statistical analysis was performed using a 2-tailed, unpaired t-test. *P<0.05. ***P<0.001

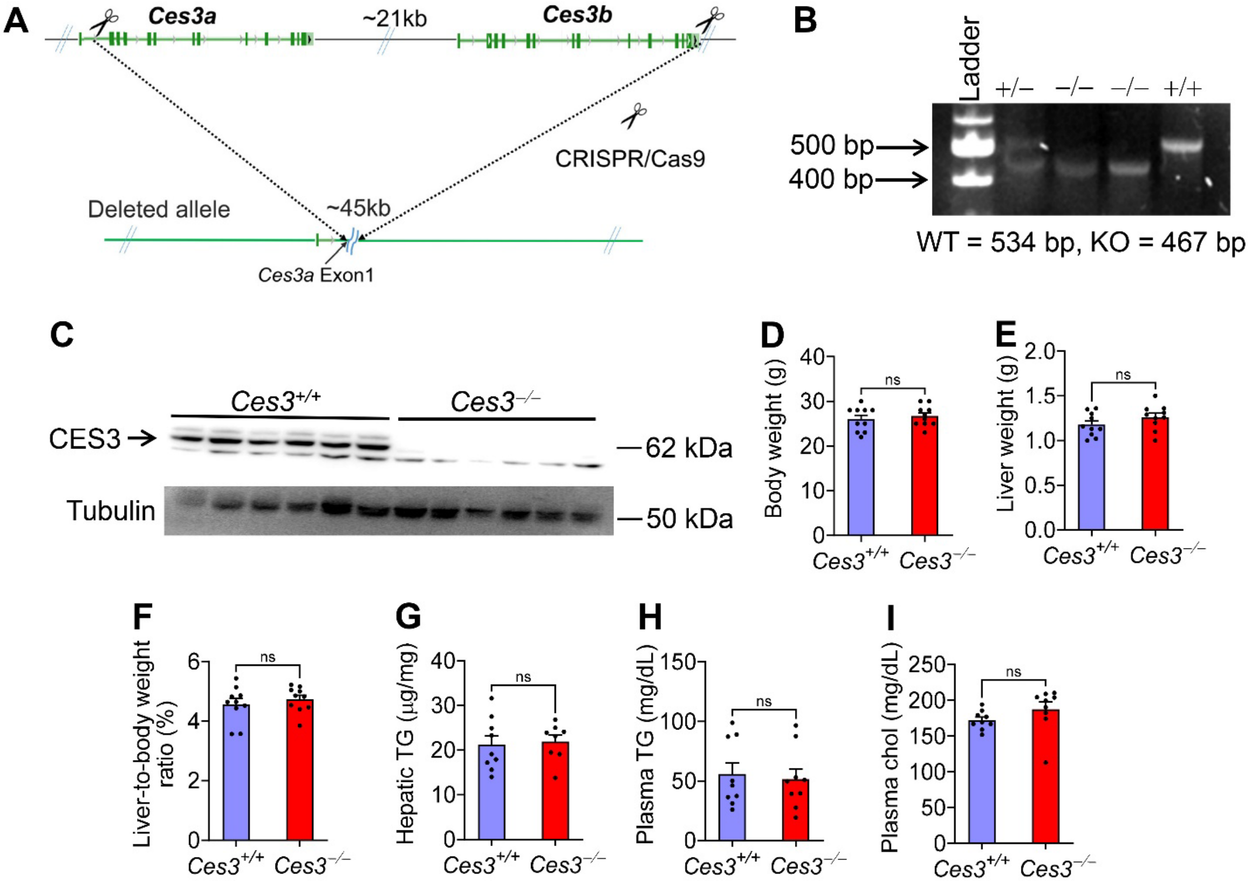

Supplementary Figure 1. Generation and characterization of *Ces3*$^{-/-}$ mice fed a regular chow diet

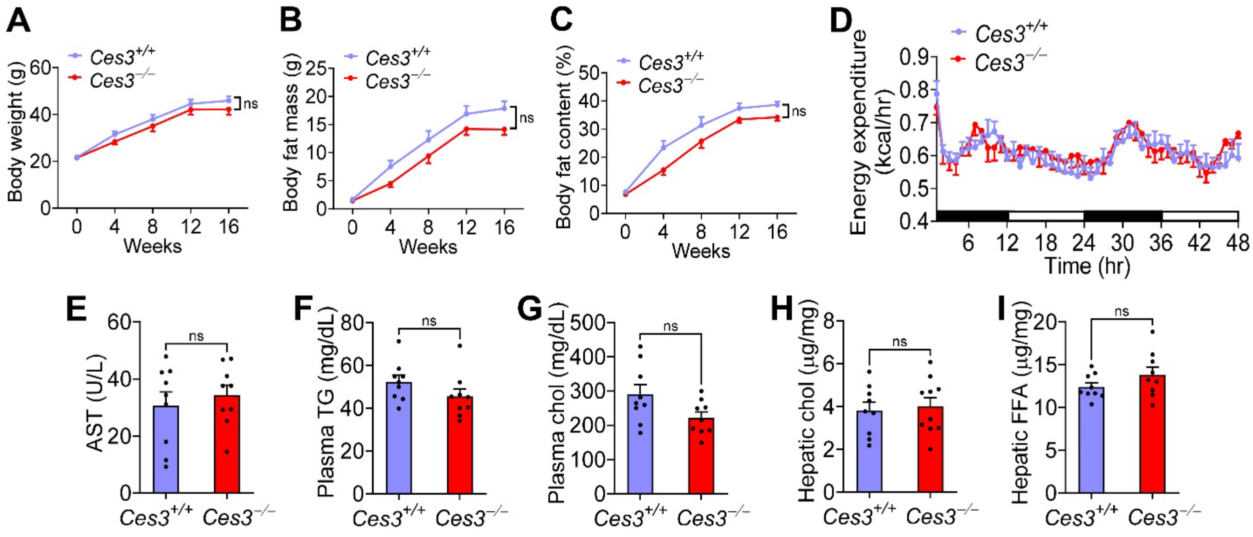

Supplementary Figure 2. Changes in Western diet-fed *Ces3*$^{-/-}$ mice

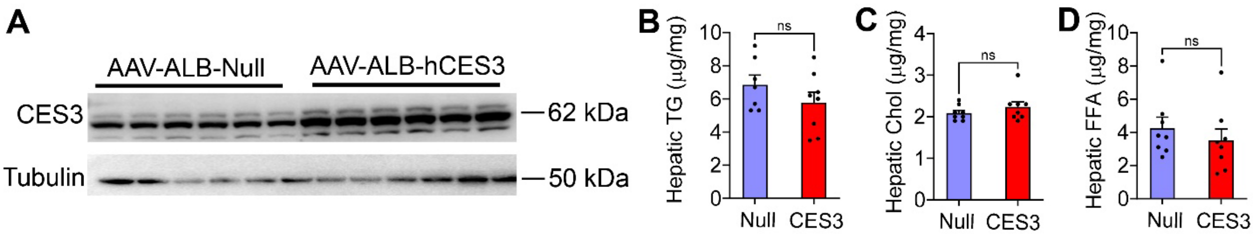

Supplementary Figure 3. Changes in regular chow diet-fed C57BL/6 mice overexpressing hepatic CES3

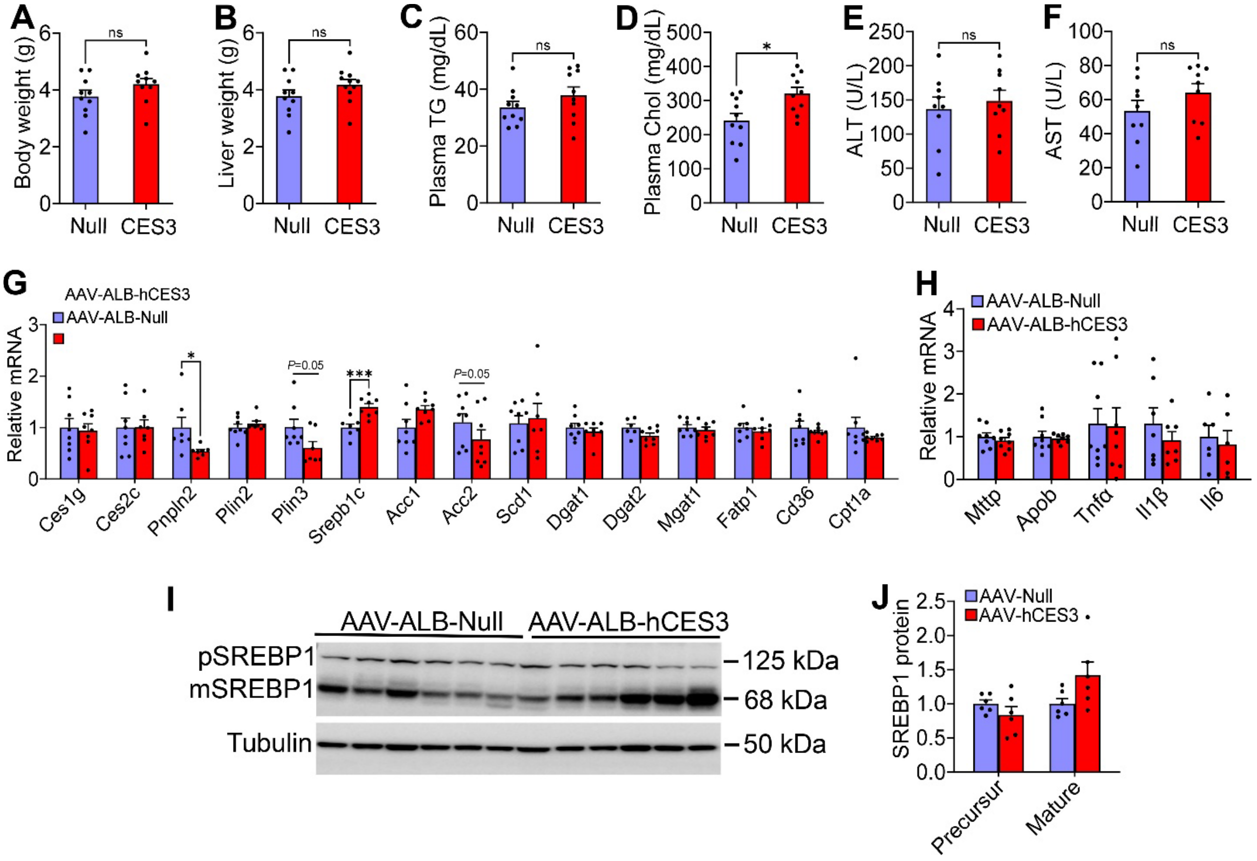

Supplementary Figure 4. Changes in Western diet-fed C57BL/6 mice overexpressing hepatic CES3
